# Supplementary material for: Expression of SARS-CoV-2 entry factors in lung epithelial stem cells and its potential implications for COVID-19
Source: Sci Rep. 2020 Oct 20;10:17772. doi: 10.1038/s41598-020-74598-5 (PMC7576138; doi:10.1038/s41598-020-74598-5)
Supplement: Supplementary file 1 — Supplementary Figures [file 41598_2020_74598_MOESM1_ESM.pdf]

Supplementary Figures

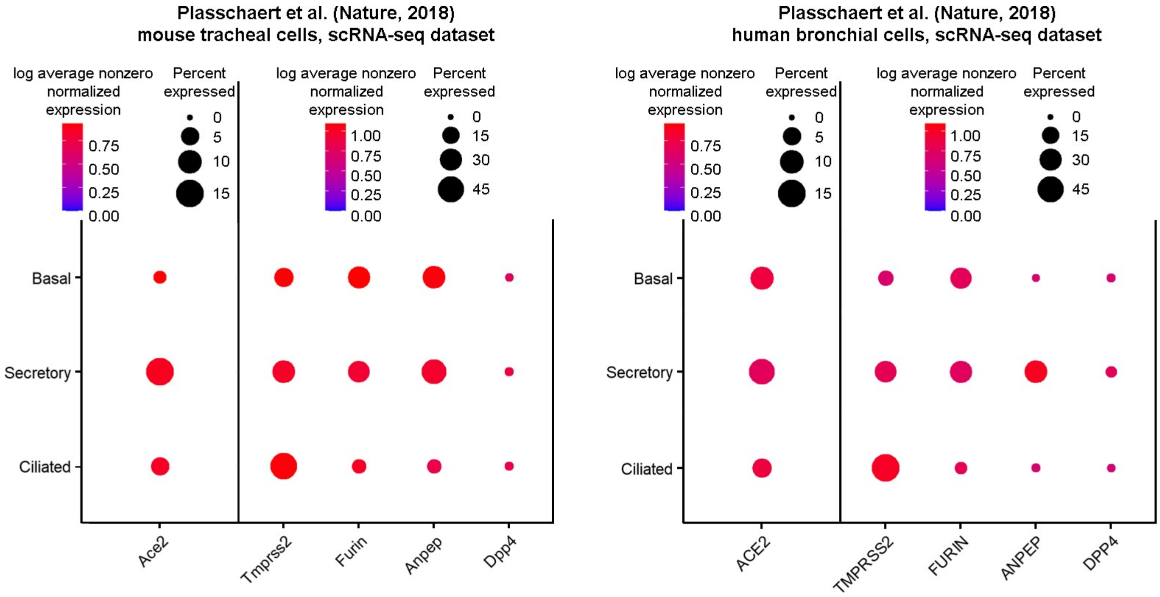

Supplementary Figure 1. Expression of the SARS-CoV-2 entry factors in mouse and human proximal airway epithelial cells: mouse trachea scRNA-seq dataset (left panel) and human bronchiole scRNA dataset (right panel)[1]. Gene expression was estimated in accordance with the cell type labels provided in the original paper. The dot size indicates the proportion of cells among the respective cell type population with greater-than-zero expression of the respective SARS-CoV-2 entry factor, while the dot color indicates the log average nonzero expression value.

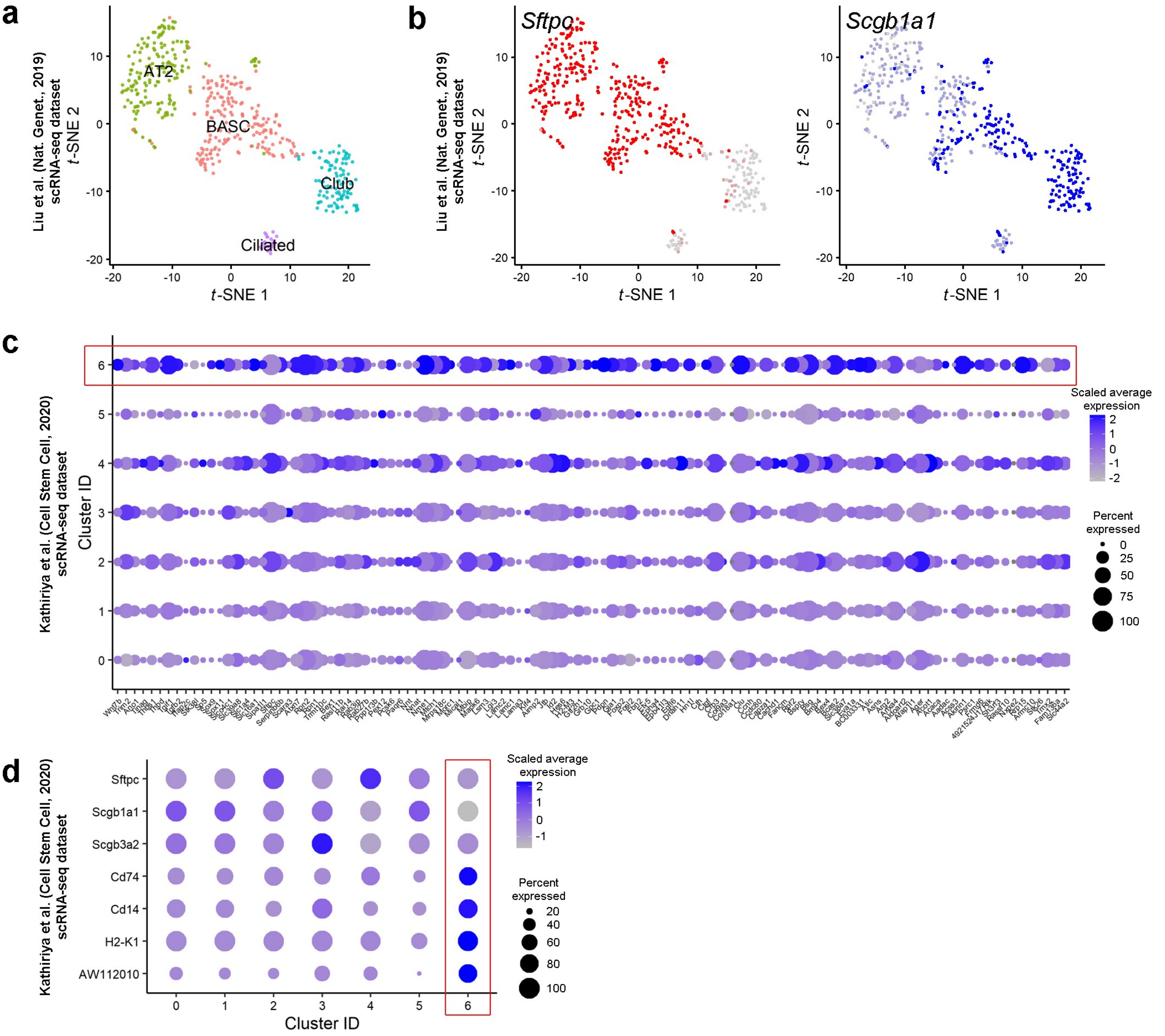

Supplementary Figure 2. Validation of cell clusters in scRNA-seq datasets. (**a**) *t*-SNE visualization of 472 scRNA-seq profiles from the scRNA-seq dataset of FACS-sorted murine epithelial cells[2], colored by cluster assignment and annotated *post hoc*. (**b**) *t*-SNE of 472 scRNA-seq profiles (points) colored by expression of representative AT2 cell and club cell markers (*Sftpc* and *Scgb1a1*, respectively). (**c**) Expression of progenitor cell markers[3] in different subpopulations (1-6) of club cells[4]. Cells in Cluster 6 cells demonstrate an elevated expression of progenitor cell markers. (**d**) Expression of lineage markers of mature club cells (*Scgb1a1* and *Scgb3a2*) and AT2 cells (*Sftpc*). Note that Cluster 6 is negative or low for these markers. The cells in this cluster are characterized by enhanced expression of *Cd14, Cd74, H2-K1*, and the lncRNA AW112010s.

References

1. Plasschaert, L. W. *et al.* A single-cell atlas of the airway epithelium reveals the CFTR-rich pulmonary ionocyte. *Nature* **560**, 377–381 https://doi.org/10.1038/s41586-018-0394-6 (2018).
2. Liu, Q. *et al.* Lung regeneration by multipotent stem cells residing at the bronchioalveolar-duct junction. *Nat. Genet.* **51**, 728–738 https://doi.org/10.1038/s41588-019-0346-6 (2019).
3. Ostrin, E. J. *et al.* β-Catenin maintains lung epithelial progenitors after lung specification. *Development* **145**, dev160788 https://doi.org/ 10.1242/dev.160788 (2018).
4. Kathiriya, J. J., Brumwell, A. N., Jackson, J. R., Tang, X. & Chapman, H. A. Distinct airway epithelial stem cells hide among club cells but mobilize to promote alveolar regeneration. *Cell Stem Cell* **26**, 346–358.e4 https://doi.org/10.1016/j.stem.2019.12.014 (2020).
