# Supplementary material for: Expression of SARS-CoV-2 entry factors in lung epithelial stem cells and its potential implications for COVID-19
Source: Sci Rep. 2020 Oct 20;10:17772. doi: 10.1038/s41598-020-74598-5 (PMC7576138; doi:10.1038/s41598-020-74598-5)
Supplement: Supplementary file 2 — Supplementary Table [file 41598_2020_74598_MOESM2_ESM.pdf]

Supplementary Table 1. RNA-seq datasets used in this study.

Analyzed Data	Identifier	Reference
scRNA-seq of mouse tracheal epithelium: basal, club, ciliated, and other rare cells (7193 3′ scRNA-seq cells + 301 full-length scRNA-seq cells)	GSE103354	[1]
scRNA-seq of mouse tracheal epithelium (7661 cells from uninjured tissue) & scRNA-seq of differentiated human bronchial epithelial cells (2970 cells)	GSE102580	[2]
bulk RNA-seq of sorted mouse AEP and AT2 cells (2 and 3 replicates per cell type)	GSE97055	[3]
scRNA-seq of sorted mouse AT1 cells (at P3, P15, P60 stages; 3149, 2940, and 1337 cells) and AT2 cells (P60; 2093 cells)	GSE106960	[4]
bulk RNA-seq of sorted mouse BASCs, AT2, and club cells (2 replicates per cell type)	GSE129440	[5]
scRNA-seq of mouse BASC, AT2, and club + ciliated cells (480 cells)	GSE118891	[6]
scRNA-seq of distal adult murine lung airway epithelium: H2-K1^{high} cells within club-like cells (3278 cells after filtering)	GSE129937	[7]

References

1. Montoro, D. T. *et al.* A revised airway epithelial hierarchy includes CFTR-expressing ionocytes. *Nature* **560**, 319–324 https://doi.org/10.1038/s41586-018-0393-7 (2018).

2. Plasschaert, L. W. *et al.* A single-cell atlas of the airway epithelium reveals the CFTR-rich pulmonary ionocyte. *Nature* **560**, 377–381 https://doi.org/10.1038/s41586-018-0394-6 (2018).
3. Zacharias, W. J. *et al.* Regeneration of the lung alveolus by an evolutionarily conserved epithelial progenitor. *Nature* **555**, 251–255 https://doi.org/10.1038/nature25786 (2018).
4. Wang, Y. *et al.* Pulmonary alveolar type I cell population consists of two distinct subtypes that differ in cell fate. *Proc. Natl. Acad. Sci. U. S. A.* **115**, 2407–2412 https://doi.org/10.1073/pnas.1719474115 (2018).
5. Salwig, I. *et al.* Bronchioalveolar stem cells are a main source for regeneration of distal lung epithelia in vivo. *EMBO J.* **38**, e102099 https://doi.org/10.15252/embj.2019102099 (2019).
6. Liu, Q. *et al.* Lung regeneration by multipotent stem cells residing at the bronchioalveolar-duct junction. *Nat. Genet.* **51**, 728–738 https://doi.org/10.1038/s41588-019-0346-6 (2019).
7. Kathiriya, J. J., Brumwell, A. N., Jackson, J. R., Tang, X. & Chapman, H. A. Distinct airway epithelial stem cells hide among club cells but mobilize to promote alveolar regeneration. *Cell Stem Cell* **26**, 346–358.e4 https://doi.org/10.1016/j.stem.2019.12.014 (2020).
